# The effects of bovine colostrum supplementation on in vivo immunity following prolonged exercise: a randomised controlled trial

**DOI:** 10.1007/s00394-017-1597-6

**Published:** 2017-12-22

**Authors:** A. W. Jones, D. S. March, R. Thatcher, B. Diment, N. P. Walsh, Glen Davison

**Affiliations:** 1 Lincoln Institute for Health, University of Lincoln, Lincoln, UK; 2 Department of Infection, Immunity and Inflammation, College of Life Sciences, University of Leicester, Leicester, UK; 3 Institute of Biological, Environmental and Rural Sciences, Aberystwyth University, Aberystwyth, UK; 4 Cambridge Centre for Health Services Research, Department of Public Health and Primary Care, University of Cambridge, Cambridge, UK; 5 Extremes Research Group, College of Health and Behavioural Sciences, Bangor University, Bangor, UK; 6 Endurance Research Group, School of Sport and Exercise Sciences, University of Kent at Medway, Chatham, ME4 4AG UK

**Keywords:** Running, Host defence, Contact hypersensitivity, Diphenylcyclopropenone, Whole integrated immune response

## Abstract

**Background:**

Bovine colostrum (COL) has been advocated as a nutritional countermeasure to exercise-induced immune dysfunction, but there is a lack of research with clinically relevant in vivo measures.

**Aim:**

To investigate the effects of COL supplementation on in vivo immunity following prolonged exercise using experimental contact hypersensitivity (CHS) with the novel antigen diphenylcyclopropenone (DPCP).

**Methods:**

In a double-blind design, 31 men were randomly assigned to COL (20 g/day) or placebo (PLA) for 58 days. Participants ran for 2 h at 60% maximal aerobic capacity on day 28 and received a primary DPCP exposure (sensitisation) 20 min after. On day 56, participants received a low-dose-series DPCP challenge to elicit recall of in vivo immune-specific memory (quantified by skinfold thickness 24 and 48 h later). Analysis of the dose–response curves allowed determination of the minimum dose required to elicit a positive response (i.e., sensitivity).

**Results:**

There was no difference in summed skinfold thickness responses between COL and PLA at 24 h (*p* = 0.124) and 48 h (*p* = 0.405). However, sensitivity of in vivo immune responsiveness was greater with COL at 24 h (*p* < 0.001) and 48 h (*p* = 0.023) with doses ~ twofold greater required to elicit a positive response in PLA.

**Conclusions:**

COL blunts the prolonged exercise-induced decrease in clinically relevant in vivo immune responsiveness to a novel antigen, which may be a mechanism for reduced illness reports observed in the previous studies. These findings also suggest that CHS sensitivity is highly relevant to host defence.

## Introduction

Upper respiratory tract symptoms (URS) are the most common ailment reported by athletes to medicine clinics at major sporting events [1–3]. It has long been considered that transient perturbations in host immunity following strenuous training and/or competition increase susceptibility to infectious (pathogenic) causes of URS [4]. Numerous studies have shown significant decreases in circulating and mucosal immune function in individuals undergoing heavy physical exertion [5]. The majority of these studies have relied on in vitro measures of immune function where the clinical relevance of such investigations is often questioned [6]. The importance of in vivo measures of immunity to determine the clinical relevance of a change in immune function in an exercise context has been recognised [7]. The clinical relevance of such measures is further supported in immune-deficient populations (e.g., HIV-positive children), whereby the degree of responses relates to relevant clinical outcomes (respiratory infections and mortality) [8–10].

Topical skin exposure to novel antigens (contact sensitisation) such as diphenylcyclopropenone (DPCP) allows for the effects of systemic stressors on the induction and elicitation phases of in vivo T-cell-mediated immune response to be quantified by oedema and erythema [11–13]. By use of this model of experimental contact sensitisation, Harper-Smith et al. [14] demonstrated that participation in a single bout of prolonged (2 h) moderate exercise compared to rest significantly reduced both the induction and the elicitation of in vivo cell-mediated immunity. Recent work demonstrating that the same exercise stressor does not impair responses to a skin irritant [15] has further established this as a controllable, reproducible, and valid marker of exercise-induced immunity (antigen T-cell-mediated responses). This rigorous model with control of both the dose and timing of the sensitisation to the novel antigen permits the investigation of strategies to counter the effects of prolonged exercise on immunity.

Several nutritional interventions have been proposed as countermeasures to exercise-induced immune dysfunction. Oral supplementation of bovine colostrum (COL) reduces the episode incidence and days of URS in adults involved in exercise training by a magnitude that is greater than the smallest clinically important difference [16]. The mechanism(s) behind such effects remain unclear, but it has been proposed that it is linked to COL reducing perturbations in cellular immunity following prolonged exercise [17]. We have previously demonstrated that COL blunts the decrease in blood neutrophil effector functions following prolonged exercise [18, 19]. Shing et al. [20] observed a prevention of a post-exercise decrease in cytotoxic/suppressor T cells during an intensive period of training. Although such in vitro or ex vivo measures are considered sufficiently reliable and sensitive markers of immunity [7], it is difficult to conclude with any degree of certainty that modulations in these parameters alone are responsible for the alteration observed in susceptibility to URS. The cutaneous measures of immunity such as contact hypersensitivity (CHS) represent an integrated and highly coordinated (in vivo) immune response to a challenge induced by a novel antigen [21].

To our knowledge, no studies have assessed the effects of COL on the in vivo immune response to a novel antigen following prolonged exercise. Therefore, the aim of this study was to investigate the effects of COL supplementation on the induction of a cell-mediated response to DPCP following prolonged exercise. We hypothesised that COL supplementation would blunt prolonged exercise-induced decreases in the induction of in vivo immunity. Although not on the World Anti-Doping Agency’s list of banned substances, supplementation of COL is not recommended by the governing body, because it contains growth factors such as insulin-like growth factor I (IGF-I) that “may influence the outcome of anti-doping tests” [22]. Given that this study may have clinically relevant implications for athletes, the secondary aim was to investigate whether COL caused any unwarranted changes in IGF-I concentrations.

## Materials and methods

### Trial design

This double-blind randomised placebo controlled trial was approved by the Aberystwyth University Research Ethics Committee and all experimental procedures were conducted in line with the Declaration of Helsinki. All participants provided both verbal and written consent following information on experimental procedures. All laboratory visits involving exercise also required completion of a physical activity readiness questionnaire (PAR-Q).

### Participants

**Fig. 1:**
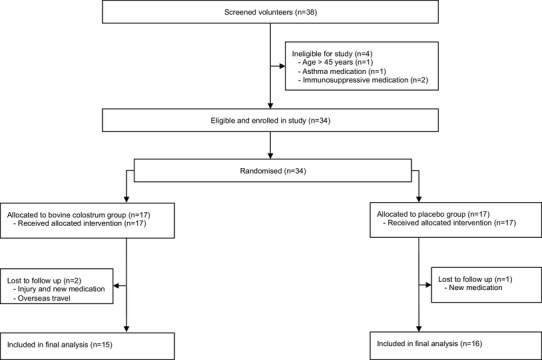
Schematic diagram of study design

Thirty-four participants were eligible for the present investigation (Fig. 1). Participants were included if they were male, healthy (as determined by PAR-Q), recreationally active, non-smokers, and aged 18–45 years. Exclusion criteria were use of regular medication or dietary supplements, known allergy or intolerances to milk products, eczema or dermatitis on the upper arm or lower back, regular exposure to ultraviolet radiation, tendency for abnormal scarring (e.g. keloid), previous contact with DPCP, participation in other research studies that involved skin patch testing, or blood donation or infection 4 weeks prior to the study.

### Supplementation

**Fig. 2:**
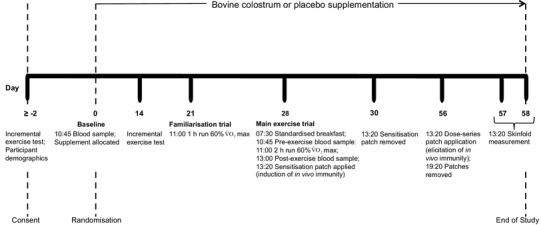
Flowchart of study participants

Participants were randomly assigned (using a computerised list generated at randomiser.org by a researcher not involved in data collection) to a COL group (*n* = 17) or placebo (PLA) group (*n* = 17) following stratification on age and aerobic fitness (determined in first incremental exercise test). COL and PLA powdered supplements were provided in sealed sachets that did not identify the contents to a study investigator (blinded to group allocation) for distribution to participants. Participants consumed 20 g per day (10 g morning and evening: mixed with 250–300 mL of water and consumed on an empty stomach) of COL or an isoenergetic/isomacronutrient PLA (skimmed milk powder and milk protein concentrate) for 58 days (28 days before and after the main exercise trial, and the 2 days of elicitation) (Fig. 2).

### Preliminary visits and standardisation

Participants performed a continuous incremental test (1 km h^− 1^ min^− 1^) following 3 min at 7 km h^− 1^ to volitional exhaustion on a treadmill with a 1% grade (PPS 55med, Woodway GmbH, Weil am Rhein, Germany). Expired gas was analysed by an online breath-by-breath gas analysis system (Jaeger Oxycon Pro, Hoechberg, Germany). Strong verbal encouragement was provided in the later stages of the test to encourage maximal effort. Maximal oxygen uptake ($\dot {V}{{\text{O}}_2}$ max) was determined as the highest 30 s average during the test.

At least 48 h following the incremental test and after an overnight fast (from midnight), participants reported to the laboratory at 10:30 for a blood sample (Baseline, day 0) prior to commencing supplementation. Fourteen days into the supplementation period, $\dot {V}{{\text{O}}_2}$ max of participants was re-tested. Seven days later, participants performed a familiarisation trial consisting of a 1 h run to habituate with study procedures and verify speed equivalent to 60% $\dot {V}{{\text{O}}_2}$ max.

For the 24 h prior to the main experimental trial, participants were provided a standardised diet of 60% (energy from) carbohydrate: ~ 5.4 g kg^− 1^ body mass (BM); 25% fat: ~ 1.0 g kg^− 1^ BM; 15% protein: ~ 1.3 g kg^− 1^ BM and water: 35 mL kg^− 1^ BM [23]. This diet matched the estimated daily energy expenditure requirements for each participant estimated by the equation of Harris and Benedict [24] multiplied by a physical activity factor of 1.5 (note: relative rest was required on this day). This diet did not include any caffeine or alcohol, and participants were also asked to avoid any exercise during this period. On the day of the main trial, participants were provided a standardised breakfast at 07:30 [total energy: 7.5 kcal kg^− 1^ BM, carbohydrate: ~ 1 g kg^− 1^ BM (60%), fat: ~ 0.2 g kg^− 1^ BM (25%) and protein: ~ 0.2 g kg^− 1^ BM (15%)]. They remained in the lab for 3 h (during which a bolus of water equivalent to 5 mL kg^− 1^ BM was provided). To further standardise dietary intake, participants were provided with a lunch prior to departure on the day of the main exercise trial [total energy: 5 kcal kg^− 1^ BM, carbohydrate: ~ 0.6 g kg^− 1^ BM (50%), fat: ~ 0.2 g kg^− 1^ BM (34%), and protein: ~ 0.2 g kg^− 1^ BM (16%)].

### Experimental procedures

#### Main exercise trial

A resting blood sample (pre-exercise) was collected at 10:45 before the 2 h run at 60% $\dot {V}{{\text{O}}_2}$ max commenced at 11:00. All participants were permitted diluted cordial (four volumes of water to 1 volume of sugar-free cordial at 2 mL kg^−1^ of BM) every 15 min during the exercise but not at end of the exercise. Expired gas (2 min) was analysed with measurements commencing at 30, 60, and 90 min of exercise (Jaeger Oxycon Pro, Hoechberg, Germany). Heart rate (Polar S610, Polar Electro Oy, Kempele, Finland) and rating of perceived exertion (RPE: Borg scale [25]) were monitored and recorded every 15 min during the protocol. A venous blood sample was collected immediately post-exercise (post-exercise). Participants showered (without the use of body washes or shower gels) and returned to the laboratory within 20 min of exercise completion.

#### Induction of contact sensitivity

Participants were sensitised at 13:20 using a single patch (22.8 µL of 0.125% DPCP in acetone, 30 µg cm^− 2^ DPCP) applied to the mid-lower back in accordance with the previous studies [14, 15, 21]. Following application, the patch remained in place for exactly 48 h. Participants were instructed to avoid alcohol and exercise during this period.

#### Elicitation of immune memory

Exactly 28 days following the induction of immune-specific memory (sensitisation), and in accordance with the previous studies [14, 15, 21], participants reported to the laboratory for a series of DPCP patches (10 µL of 0.0048%, 1.24 µg cm^− 2^; 0.0076%, 1.98 µg cm^− 2^; 0.0122%, 3.172 µg cm^− 2^; 0.01953%, 5.08 µg cm^− 2^; 0.03125%, 8.12 µg cm^− 2^; and 0%, 100% acetone) to be applied to the volar aspect of their right upper arm, at the same time (13:20). To minimise anatomical variability, these patches were applied in a randomly allocated order (matched between groups). All patches were removed after exactly 6 h.

#### Assessment of cutaneous responses

Participants returned to the laboratory 24 and 48 h following the application of patches for cutaneous responses (oedema, by skinfold thickness) to be measured, in triplicate, using modified skin fold callipers (Baty, West Sussex, UK) in accordance with the previous studies [14, 15, 21]. During this 48 h period, and the 24 h prior to application of the patches, participants were requested to avoid any exercise and alcohol. The dose–response curves were used to conduct sensitivity analyses to determine the minimum DPCP dose required to elicit a (positive) response. Summed increases in skinfold thickness were determined by adding values for all doses.

### Blood sampling

Participants remained seated, performing minimal movement for 10 min prior to each blood sample with the exception of immediately post-exercise, which was drawn within a few min of exercise cessation. Blood samples were collected by venepuncture [21 gauge precision needle (Becton–Dickinson, Oxford, UK)] from an antecubital vein into a vacutainer (Becton–Dickinson, Oxford, UK) containing tripotassium ethylene diamine tetraacetic acid (K_3_EDTA). A small aliquot was taken from the K_3_EDTA tube for whole blood measures, whilst the remaining sample was centrifuged at 1500*g* for 10 min at 4 °C. Ailquots of plasma were stored at − 80 °C for later analysis.

### Blood analysis

Leukocyte counts (ABX Pentra 60 C+, Horiba Medical, Montpellier, France) were determined on K_3_EDTA treated whole blood. Glucose and lactate were determined on EDTA plasma (Biosen C-Line, EKF Diagnostic, London, UK). IGF-I concentration was quantified in duplicate pre-treated (to release IGF-I from binding proteins) plasma samples at baseline and pre-exercise (4 weeks following supplementation) via a commercially available enzyme-linked immunosorbent assay (R&D Systems, Abingdon, UK).

### Statistical analysis

Sample size estimation: It was not possible to use previous data to perform power calculations, since there are no other studies that have used the present methods to assess in vivo immune function with this supplement. However, we have undertaken a number of studies on various immune markers and, on average, have observed a large effect (Cohen’s *d* = 1.25) with the dosage of bovine colostrum used in the present study (e.g., [18, 19, 26]). To detect such a magnitude of effect in this study, a sample size of *n* = 15 per group (*n* = 30 in total) is required to provide > 90% power.

Data shown in the text, tables, and figures are presented as mean ± standard deviation unless stated otherwise. Statistical analysis of the dose–response curves was performed using Graph Pad Prism version 7 (GraphPad, San Diego, USA). Statistical analyses of all blood measures were performed via SPSS (v22.00; SPSS Inc., Chicago, IL, USA). If possible, data not normally distributed were normalised with log or square root transformation before further analysis; otherwise, non-parametric tests were used. A two-factor mixed ANOVA (group × time) was carried out on blood leukocytes, plasma glucose, lactate, and IGF-I. Any significant main effects in the ANOVA were further analysed by post hoc two-tailed paired or independent *t* tests with Holm–Bonferroni correction. Independent *t* tests were used to compare HR, oxygen uptake (%$\dot {V}{{\text{O}}_2}$ max, $\dot {V}{{\text{O}}_2}$), RPE, and summed skinfold thickness (at 48 h). Mann–Whitney *U* test was used to assess summed skinfold thickness responses to DPCP at 24 h. Statistical significance was accepted at *p* < 0.05.

## Results

### Participants

From initially screened and included volunteers, 31 participants were analysed in this trial (Fig. 1). Follow-up was not possible in two participants from COL group due to injury (outside of experimental protocol which also required acute anti-inflammatory treatment) and overseas travel leading to prolonged period where supplement was not consumed. One participant from PLA group was excluded following a change in circumstances (prescribed medication), meaning that they no longer met the eligibility criteria. Groups remained well matched with no significant differences in physical characteristics (Table 1).

**Table 1:** Participant characteristics

	COL	PLA	*p* value
Age (years)	23.4 ± 4.7	25.3 ± 5.5	0.307
Body mass (kg)	76.4 ± 9.2	73.7 ± 8.1	0.388
Height (m)	1.81 ± 0.05	1.79 ± 0.07	0.352
$\dot {V}{{\text{O}}_2}$ max (mL min^− 1^)	4272 ± 410	4152 ± 529	0.489
$\dot {V}{{\text{O}}_2}$ max (mL kg^− 1^ min^− 1^)	56.6 ± 8.0	56.4 ± 5.3	0.962

### Physiological responses to exercise

There was no significant difference in absolute (*p* = 0.979) or relative (*p* = 0.423) trial $\dot {V}{{\text{O}}_2}$ between the COL (2646 ± 254 mL min^− 1^; 61.4 ± 2.3% $\dot {V}{{\text{O}}_2}$ max) and PLA groups (2649 ± 329 mL min^− 1^; 62.3 ± 3.3% $\dot {V}{{\text{O}}_2}$ max). Similar mean HR (COL, 150 ± 14 bpm; PLA, 150 ± 11 bpm; *p* = 0.910) and RPE (COL, 13.0 ± 1.0; PLA, 12.6 ± 1.2; *p* = 0.365) values were observed during the main exercise trials. There was no significant time (*p* = 0.502), group (*p* = 0.319), or interaction effect (*p* = 0.633) for plasma glucose (COL, pre-exercise: 4.5 ± 0.4 mmol L^− 1^, post-exercise: 4.7 ± 0.7; PLA, pre-exercise: 4.4 ± 0.6, post-exercise: 4.4 ± 0.6). There was a significant time effect for plasma lactate (*p* < 0.001) with a significant increase from pre-exercise (*p* < 0.001) to post-exercise, but there was no group (*p* = 0.804) or interaction effect [*p* = 0.506 (COL, pre-exercise: 1.4 ± 0.4 mmol L^− 1^, post-exercise: 1.9 ± 0.5; PLA, pre-exercise: 1.3 ± 0.2, post-exercise: 2.0 ± 0.7)].

### Immune cell counts

No significant group or interaction effects were evident for total or differential leukocyte counts (Table 2). A main effect of time was observed for all leukocytes (Table 2).

**Table 2:** Immune cell counts prior to and following prolonged running

Cell count, 10^9^ × L^− 1^	Baseline	Pre-exercise	Post-exercise	*p* values
				Group
				Time
				Interaction
Total leukocytes			^†,‡^	0.601
COL	5.5 ± 1.8	5.7 ± 1.3	11.6 ± 4.3	< 0.001*
PLA	4.8 ± 1.2	5.3 ± 0.9	12.0 ± 5.7	0.399
Neutrophils		^†^	^†‡^	0.566
COL	3.0 ± 1.7	3.2 ± 1.2	8.5 ± 4.5	< 0.001*
PLA	2.3 ± 0.8	2.9 ± 0.7	8.5 ± 5.2	0.382
Monocytes			^†,‡^	0.467
COL	0.5 ± 0.2	0.5 ± 0.1	0.9 ± 0.4	< 0.001*
PLA	0.5 ± 0.1	0.5 ± 0.1	0.9 ± 0.4	0.337
Total lymphocytes			^†,‡^	0.459
COL	1.7 ± 0.4	1.6 ± 0.4	2.2 ± 0.6	0.001*
PLA	1.8 ± 0.5	1.7 ± 0.4	2.4 ± 0.6	0.939
Neutrophil: lymphocyte		^†^	^†,‡^	0.371
COL	1.9 ± 1.7	2.1 ± 1.0	3.9 ± 1.7	< 0.001*
PLA	1.3 ± 0.4	1.8 ± 0.5	3.6 ± 2.0	0.430

*Significant main effect of time (*p* < 0.001). Post hoc (analysis for time effects): ^†^Significant difference compared to Baseline (*p* < 0.05), ^‡^Significant difference compared to pre-exercise

### Plasma IGF-I

There was a significant time effect for plasma IGF-I (*p* = 0.003), but there was no group (*p* = 0.649) or interaction effect (*p* = 0.987) [COL, baseline: 125 ± 23 ng mL^− 1^, pre-exercise (4 weeks): 135 ± 31 ng mL^− 1^; PLA, baseline: 131 ± 33 ng mL^− 1^, pre-exercise: 140 ± 33 ng mL^− 1^].

### In vivo immune responses

**Fig. 3:**
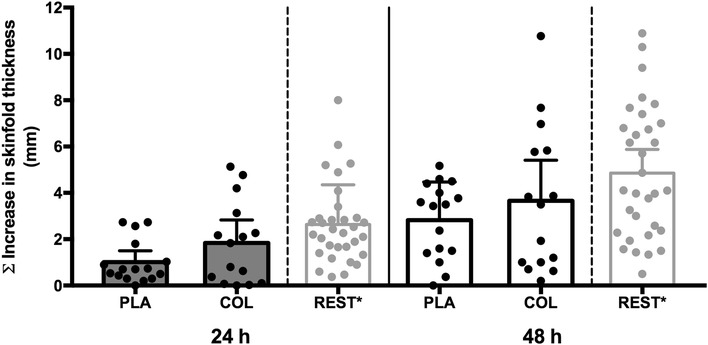
Summed increase in skinfold thickness at 24 and 48 h in response to DPCP challenge 28 days after sensitisation. Asterisk: participants of the non-exercising control arms (*n* = 32) from the previous studies [14, 15] serve as an additional comparison (i.e., 120 min of seated rest prior to sensitisation with DPCP) to demonstrate typical decreases in summed skinfold responses to DPCP with prolonged exercise. Columns indicate mean values for each group. Error bars represent 95% confidence intervals

**Fig. 4:**
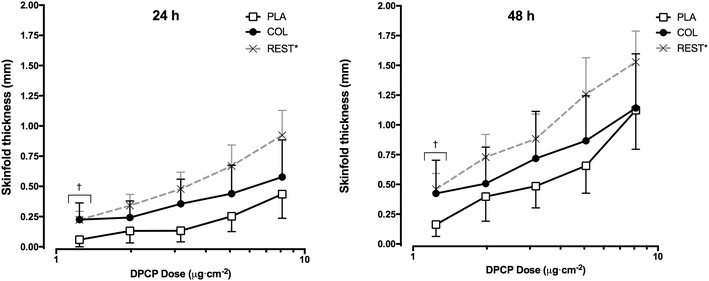
Skinfold responses to the full dose-series DPCP challenge 28 days after sensitisation. Asterisk: participants of the non-exercising control arms (*n* = 32) from the previous studies [14, 15] serve as an additional comparison (i.e., 120 min of seated rest prior to sensitisation with DPCP) to demonstrate typical decreases in sensitivity of immune-specific memory to DPCP with prolonged exercise. Points indicate mean values for each group. Error bars represent 95% confidence intervals. Dagger: significant difference between PLA and COL (*p* < 0.05)

The summed skinfold response was not significantly different between groups at 24 h (COL: 1.84 ± 1.79 mm, PLA: 1.01 ± 0.92 mm, *p* = 0.124) or 48 h (COL, 3.61 ± 3.21 mm; PLA, 2.83 ± 1.64 mm, *p* = 0.405) (Fig. 3). Analysis of the dose–response curves allowed determination of the minimum DPCP dose required to elicit a positive response (i.e., sensitivity) (Fig. 4). The minimum dose required at 24 h was 0.4 and 0.8 µg cm^− 2^ for COL and PLA groups, respectively (*p* < 0.001), indicating that a dose of 2.0-fold greater was required to elicit a positive response in the PLA group. At 48 h, the threshold was 0.4 and 0.7 µg cm^− 2^ for COL and PLA groups, respectively (*p* = 0.023), indicating that a dose of 1.8-fold greater was required to elicit a positive response in the PLA group. Direct comparisons between groups at each dose revealed a greater skinfold thickness response in COL compared to PLA for the lowest DPCP dose at both 24 h (COL: 0.22 ± 0.25 mm, PLA: 0.06 ± 0.11 mm, *p* = 0.011) and 48 h (COL: 0.42 ± 0.50 mm, PLA: 0.16 ± 0.19 mm, *p* = 0.048).

## Discussion

The aim of this study was to investigate the effects of COL supplementation on the induction of in vivo immune responses to a novel antigen following prolonged exercise. The previous evidence suggests that completion of prolonged exercise prior to sensitisation to the novel sensitising chemical, DPCP, can impair the induction of antigen-specific memory [14]. This provides a robust method to evaluate the effects of purported nutritional countermeasures on exercise-induced immune dysfunction in vivo. COL did not significantly affect the overall summed skinfold response to DPCP, but COL supplementation induced greater sensitivity of antigen-specific memory recalled 4 weeks following the initial sensitisation. A greater contact hypersensitivity response to the lowest DPCP dose was also evident in the COL group, but there was no difference at the higher doses.

The findings of the present study extend our current knowledge on the benefits of COL as an immune-enhancing supplement in humans. Evidence, to date, suggests that the effects of COL may be more apparent in the early recovery period following prolonged exercise [18, 19]. However, the immunoprotective effects of COL (within a model of exercise-induced immune dysfunction) thus far in humans have only been demonstrated with in vitro measures of blood neutrophil function. Although investigations on whole blood maintains the proximity of leukocytes and the extracellular milieu of leukocytes compared to other in vitro measures of immune function, the use of in vivo measures is considered more clinically relevant [7, 11, 22]. We previously demonstrated that COL limits the increased salivary bacterial load in physically active males during the winter months [26], and proposed this to be a relevant marker of in vivo (innate) immunity that needed further testing in an exercise immunology context. Here, we have used a controllable, reproducible, and valid marker of exercise-induced immunity in vivo.

With regard to the overall reactivity to DPCP, we did not observe any significant effects of COL. In the one previous study investigating a nutritional countermeasure (carbohydrate supplementation during exercise) using this experimental model [21], there were no significant effects on either sensitivity or overall reactivity to DPCP following prolonged exercise. The previous studies [14, 15] have consistently shown that participation in prolonged exercise prior to primary sensitisation to DPCP decreases summed responses of all challenge sites and increases the minimum dose (threshold) required to elicit a positive response during recall of immune-specific memory. As a result of exercise-induced impairment in cell-mediated immunity, a three-to-fourfold greater (i.e., lower sensitivity), DPCP dose was required to evoke a positive response in the exercise group than the non-exercising control group. Similarly, when compared to the resting groups of Harper-Smith et al. [14] and Diment et al. [15], the exercise groups in this study demonstrate a reduction in summed responses (Fig. 3). However, the findings of this study demonstrate that COL acts as a nutritional countermeasure to prolonged exercise-induced decrements in the sensitivity of clinically relevant in vivo immune responsiveness to a novel antigen.

There is unequivocal evidence that a threshold effect exists for cutaneous sensitisation [27–29]. Indeed, with experimental contact dermatitis, there are threshold doses where an allergic state is not clinically elicited despite prior sensitisation [30]. In contrast, a major goal of vaccines is to sensitise host defences for future exposure to a pathogen [31]. Hence, greater immune responses by the host during exposure to low doses of such antigens are deemed to be beneficial for limiting the spread of the infectious agent. The previous findings of no inhibitory effect of prolonged exercise on skinfold responses to the irritant, croton oil, provide evidence that in the context of DPCP, responses are dependent on cell-mediated events rather than local inflammatory processes [15]. Given that a recent meta-analysis [16] of five randomised controlled trials (including one from our laboratory) of COL supplementation showed a reduction in the incidence of URS during exercise training [26, 32–34], it is likely that an increase in recall sensitivity provides a mechanistic explanation for reduced URS. It will be useful, however, for future studies to monitor URS in the period following DPCP sensitisation. It is beyond the scope of the findings of this study and the previous evidence to suggest that T-cell-mediated responses at lower doses of DPCP are of greater biological significance (i.e. risk of URS) than summed responses. Our data, however, does at least suggest that the sensitivity of cutaneous recall responses to DPCP gives a better indication of clinically relevant immunological changes following nutritional (bovine colostrum) interventions.

It must be acknowledged that the present study design does not allow us to fully establish whether the potential effect of COL on the immune system occurred during the induction and/or the elicitation of cell-mediated immune responses. Limiting consumption of COL to the 4 weeks between induction and elicitation only might have provided mechanistic insight regarding this question, but this does not reflect the application of COL in the real-world setting. Within the field of contact sensitisation, the main factor considered to determine the extent of elicitation is the strength of the induction [30]. In addition, the stronger the degree of induction, the lower the dose that sensitised individuals will react to upon any recall challenge [12]. Furthermore, the evidence that the induction phase of T-cell memory is more susceptible to the effects of prolonged exercise than the elicitation phase [14] may also point towards the period where participants would have benefited most from a nutritional countermeasure such as COL. Biswas et al. [35] did demonstrate that COL can differentially affect in vitro stimulation of human peripheral blood mononuclear cells by enhancing IFNγ production during weak antigenic stimulation but not under conditions of strong antigen stimulation.

Although T-cell infiltration plays a central role in the orchestration of CHS responses, this is initiated by the non-specific ‘sensitivity’ of other local (dendritic, Langherhans) cells that respond to perturbations induced by the antigen [36]. In addition to the induction phase, recent evidence suggests that the recruitment of antigen-primed CD8+ T cells in response to elicitation of immune memory may also depend on infiltration and chemoattractants released by inflammatory cells (e.g. neutrophils) [37, 38]. It was a limitation of the present study that we did not measure circulating cytokines or in vitro measures of immunity alongside in vivo immunity. These may have provided some insight into the mechanisms of action or the effects of COL on signals that may have triggered the migration and maturation of cells involved in the in vivo response and whether possible tissue priming effects resulted in enhanced immunosurveillance (and explain the greater responses at lower concentrations of DPCP in the COL group). Future studies should also explore the effects of COL supplementation on immune parameters prior to both induction and elicitation separately to further determine mechanisms. However, it is important to note that the primary outcome measure, CHS, represents the whole integrated in vivo immune response that is considered the most clinically relevant measure [7, 11, 22] and also that in vitro responses do not necessarily predict in vivo responses [15, 21]. CHS measurements recorded prior to the 24 h time point may also provide useful information on the improved sensitivity in future studies. If there were any modulatory effects of COL on performance or stress responses, this could also contribute indirectly to effects on the immune responses. However, the present findings of similar leukocyte trafficking, and physiological measures, between groups (along with similar findings in the previous studies that also found no difference in stress hormone responses, e.g., [18, 19]), suggest that subjects in this study were exposed to a matched exercise-induced stress, supporting the previous evidence that the effects of COL are not due to such indirect mechanisms.

This study provides further evidence that COL, as a supplementation regimen that induces clinically relevant benefits to immune health, does not increase circulating concentrations of IGF-I compared to an isoenergetic/isomacronutrient placebo. The previous evidence from one laboratory had suggested increases in IGF-I following short-periods (≤ 2 weeks) of COL supplementation [39, 40], but one of these studies was unable to confirm that it was orally administered IGF-I that appeared in the circulation (i.e., IGF-I was not absorbed from COL). An important limitation in these studies was the choice of placebo (dextrose only) as the addition of daily protein supplementation has been shown to induce similar elevations in IGF-I [41]. Subsequently, changes in circulating IGF-I following COL have not been replicated by other investigators including larger doses and longer duration of supplementation [42–45], which, in one study, was supported by no positive values in urine samples analysed within an International Olympic Committee-accredited laboratory [45]. Furthermore, in a previous study [26], we conducted a comprehensive metabolomics analysis and found no differences that would suggest COL could influence the outcome of a doping test. The balance of evidence, therefore, does not seem to support the claim that COL can increase IGF-I or influence doping tests.

## Conclusion

In summary, COL did not significantly affect the overall reactivity of in vivo response to a novel antigen but did blunt the prolonged exercise-induced decrease in sensitivity of the immune responsiveness. A difference in response to DPCP was evident at the lowest recall dose but not at the higher doses. Together with the previous findings (e.g., effects of COL on host defence and illness susceptibility), these results may also suggest that the sensitivity of CHS responses is highly relevant to host defence and in vivo protection against infection. The present study was undertaken in a fed state (to replicate real-world practices of athletes) with a recreational athlete population, and presents the first evidence of a nutritional strategy to counter exercise-induced immunodepression assessed via an established, clinically relevant in vivo marker of immunity.
